# Supplementary material for: Toward Inferring Potts Models for Phylogenetically Correlated Sequence Data
Source: Entropy (Basel). 2019 Nov 7;21(11):1090. doi: 10.3390/e21111090 (PMC7514434; doi:10.3390/e21111090)
Supplement: Supplementary file 1 [file entropy-21-01090-s001.pdf]

Supplementary Material S1: Toward Inferring Potts Models for Phylogenetically Correlated Sequence Data

Edwin Rodriguez Horta,[1,2] Pierre Barrat-Charlaix,[1,3] and Martin Weigt[1]

[1]*Sorbonne Université, CNRS, Institut de Biologie Paris-Seine, Laboratoire de Biologie Computationnelle et Quantitative – LCQB, Paris, France*
[2]*University of Havana, Physics Faculty, Department of Theoretical Physics, Group of Complex Systems and Statistical Physics, Havana, Cuba*
[3]*Biozentrum, University of Basel, Basel, Switzerland*

(Dated: November 7, 2019)

I. SUPPLEMENTARY FIGURES FOR THE MAIN TEXT

This section includes figures that are referenced from the main text.

II. RESULTS ON PROTEIN FAMILIES

We have selected 5 protein families from the data used in the mfDCA paper by Morcos at al. 2011 (Ref. [8] in the main text), cf. the table providing names, sequence lengths, sequence numbers and effective sequence numbers after standard reweighting at 80% sequence identity. These families have short sequence length for faster inference. They do use the original MSA of [8], since currently Pfam is based on reference proteomes, *i.e.* sequence ensembles are already phylogenetically pruned.

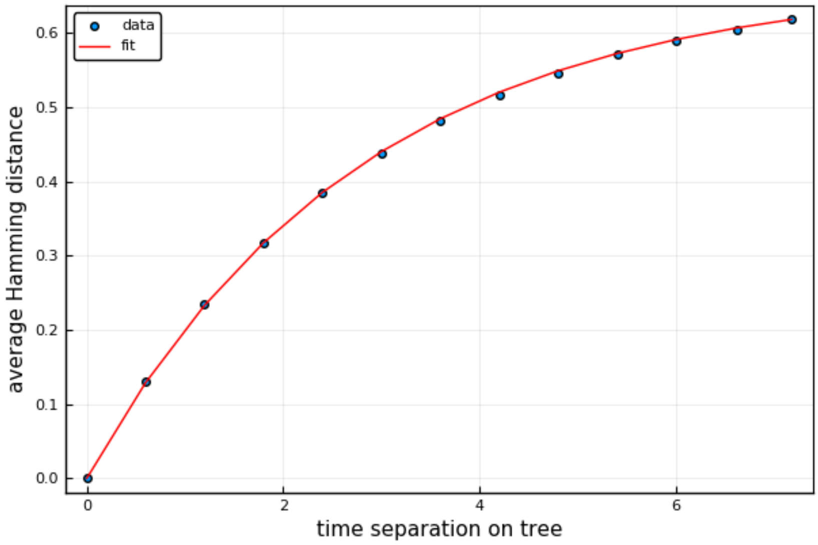

Supplementary Figure S1: Average Hamming distance between sequences as a function of their evolutionary time distance on the phylogenetic tree. Symbols are computed from artificial data with branch length $\Delta t = 0.3$ and $\mu = 0.4$. The full line shows a fit to Eq. (9), with resulting mutational parameter $\hat{\mu} = 0.357$. Note that the deviation from the true value μ results from the fact, that the dynamics of a coupled Potts model is fitted to a curve derived for an independent-site evolutionary dynamics. Slowing down due to coupling effects leads to an effectively smaller mutational rate.

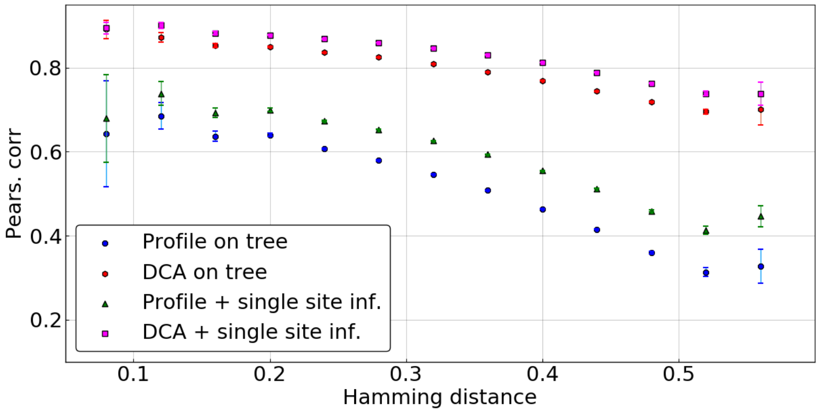

Supplementary Figure S2: Pearson correlation in predicting energies of single mutants for $\mu\, L\, \Delta t = 3$ averaged over reference sequence at a given Hamming distance to the closest sequence in the biased sample, as a function of this Hamming distance. Error bars are inversely proportional to the square root of the number of sequences in each hamming distance bin. Profile and Potts models are inferred either directly using biased data, or using corrected single site frequencies.

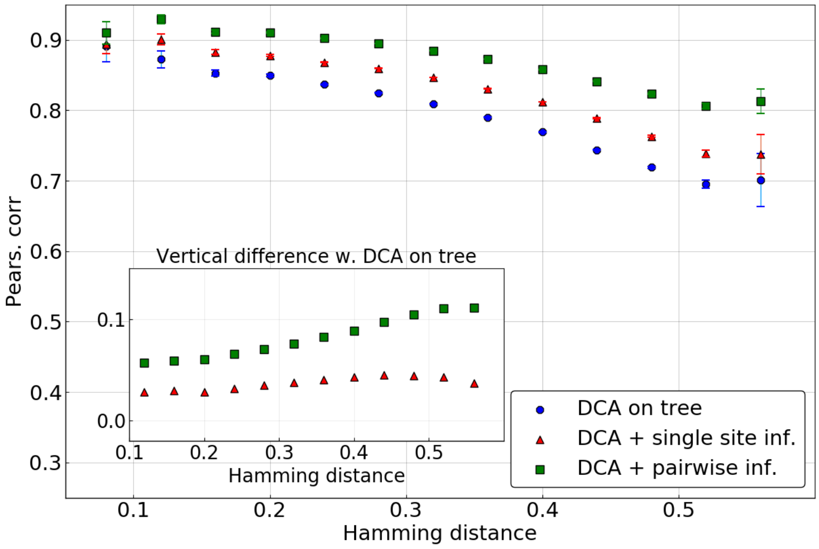

Supplementary Figure S3: Pearson correlation in predicting energies of single mutants for $\mu\, L\, \Delta t = 3$ averaged over reference sequence at a given Hamming distance to the closest sequence in the biased sample, as a function of this Hamming distance. Error bars are inversely proportional to the square root of the number of sequences in each hamming distance bin. The Potts model is inferred either directly on biased data, either using corrected single site frequencies, either using corrected pairwise frequencies.

TABLE S1: Selected protein families. Family ID, sequence length, number of sequences, number of uniqie sequences and effective number of sequences at sequence ID 0.8 are given.

Family ID	L	M	M_{unique}	M_{eff}
PF00111	52	8977	6400	3746
PF00046	57	13439	6088	1630
PF00013	57	7921	4755	2227
PF00018	48	5643	3346	1734
PF00084	56	6244	4004	2638

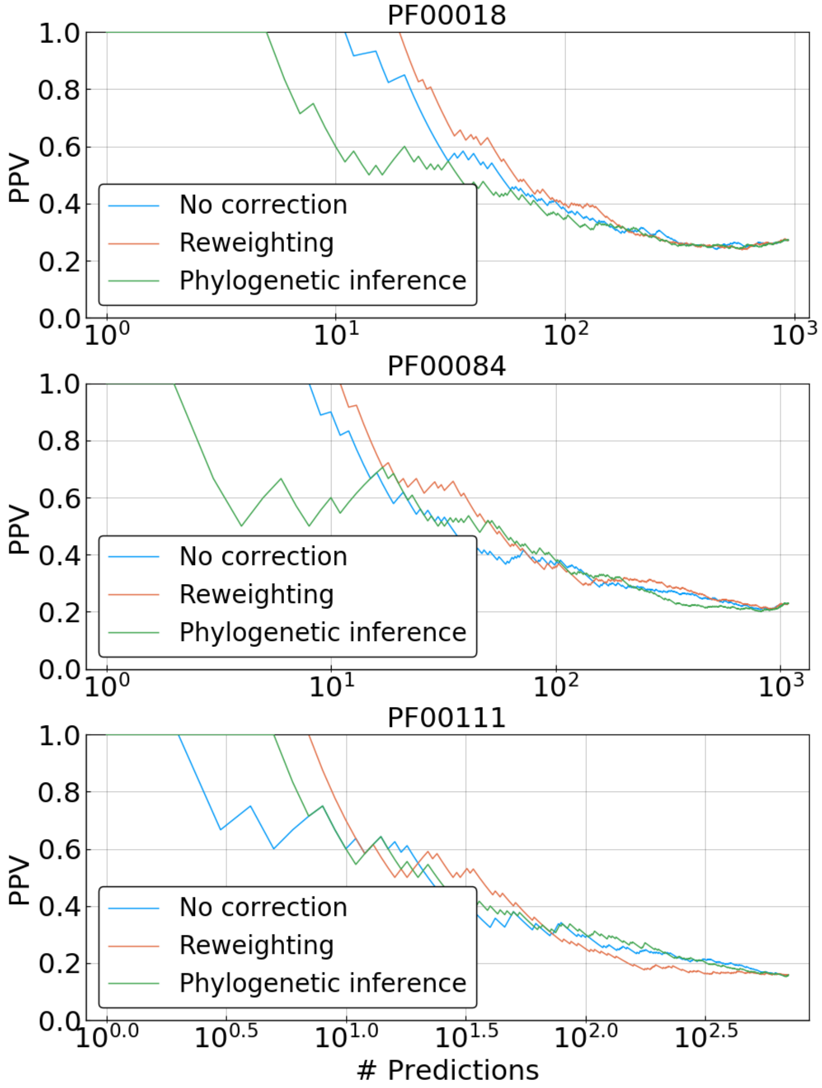

Supplementary Figure S4: Positive predictive value for predicting contacts in representative structures for the three other protein families: PF00014, PF00084 and PF00111. The blue lines indicate a naive DCA method without any correction for phylogeny. The orange lines show results for the sequence re-weighting scheme. The green lines show results after our phylogenetic inference scheme. The latter is outperformed for the top couplings in all three cases.

III. RESULTS FOR $\mu L \Delta t = 5$

This Appendix shows analogous results to those in the main text, but for $\mu L \Delta t = 5$. This means, that the branches of the phylogenetic tree are longer than before, and thus the phylogenetic effects are weaker. Nevertheless, the proposed procedure for phylogeny-based inference and correction of DCA models leads to clear improvements along all test.

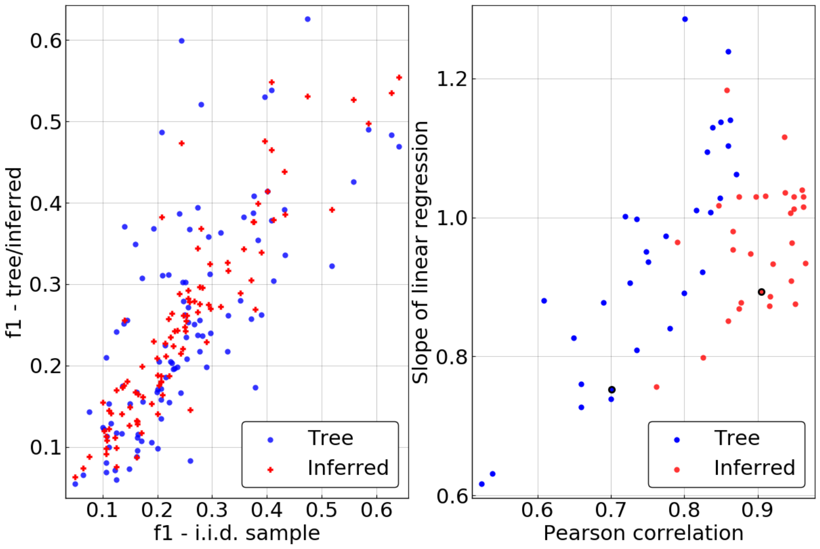

Supplementary Figure S5: Same as Fig. 3, but for $\mu L \Delta t = 5$.

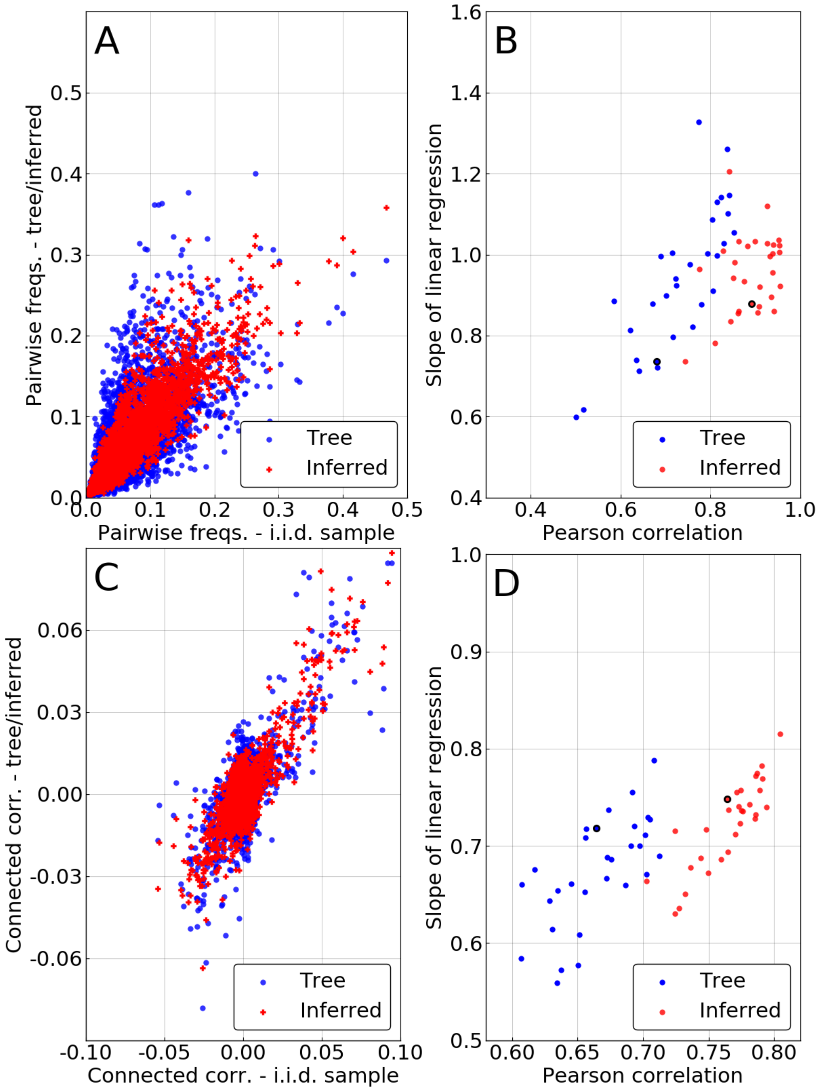

Supplementary Figure S6: Same as Fig. 4, but for $\mu L \Delta t = 5$.

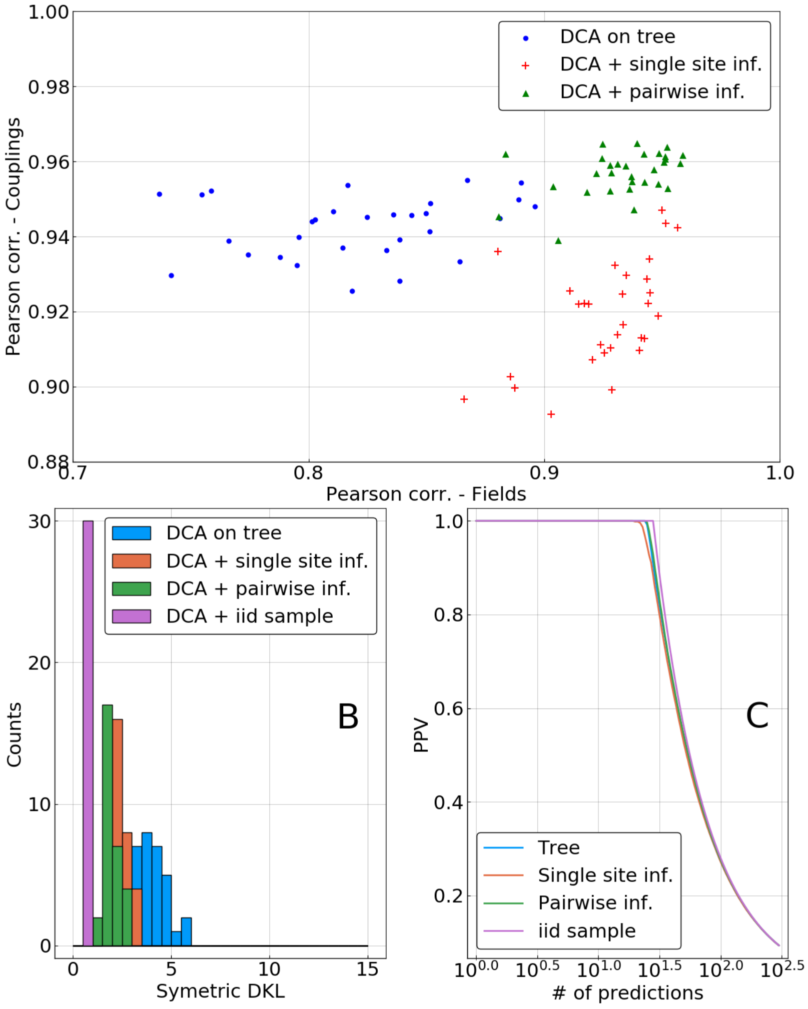

Supplementary Figure S7: Same as Fig. 5, but for $\mu L \Delta t = 5$.

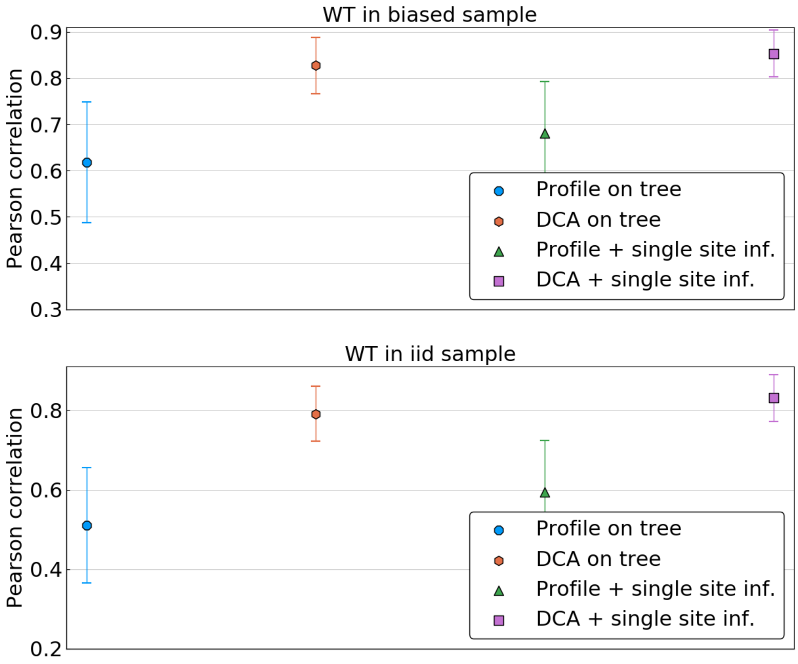

Supplementary Figure S8: Same as Fig. 6, but for $\mu L\Delta t = 5$.

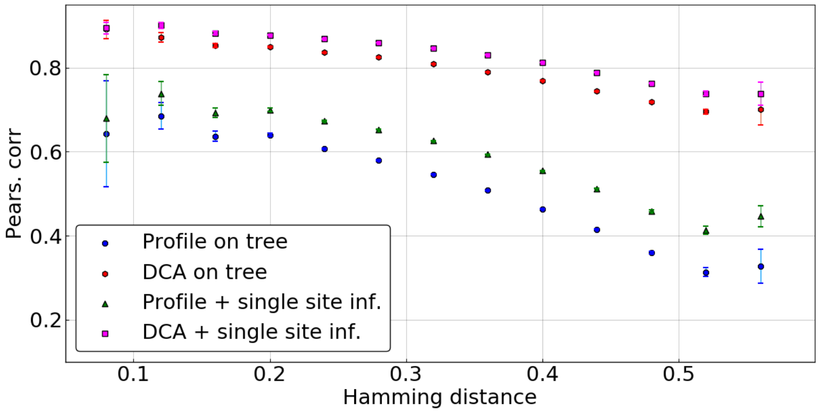

Supplementary Figure S9: Same as Supplementary Fig. S2, but for $\mu L\Delta t = 5$.

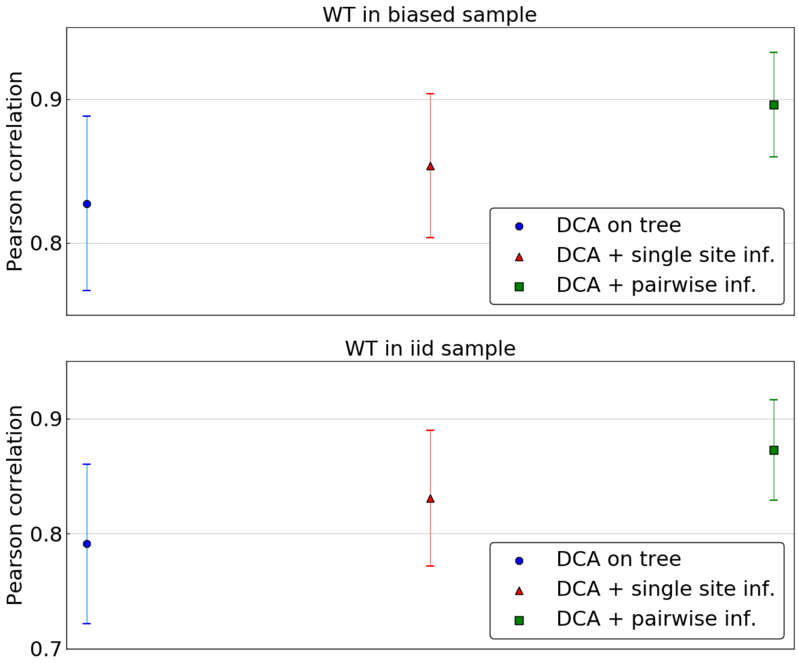

Supplementary Figure S10: Same as Fig. 7 but for $\mu L \Delta t = 5$.

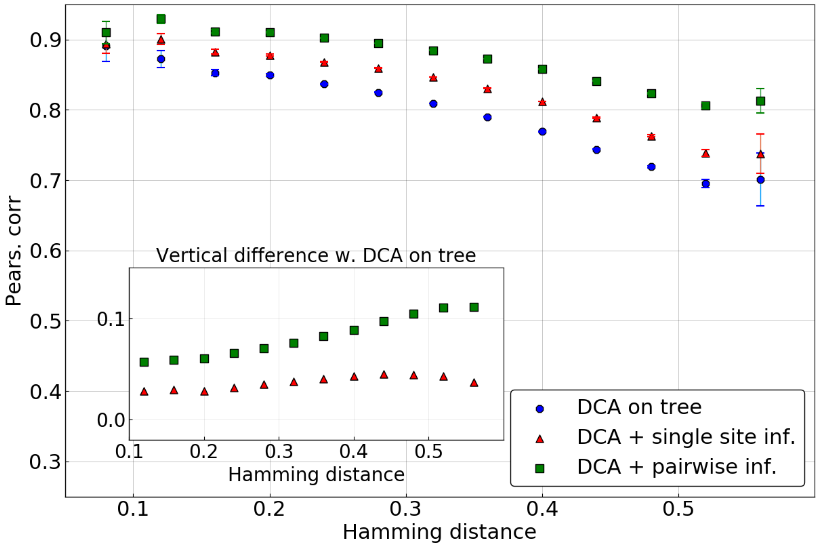

Supplementary Figure S11: Same as Supplementary Fig. S3, but for $\mu L \Delta t = 5$.
